# Cardiopulmonary Exercise Capacity and Preoperative Markers of Inflammation

**DOI:** 10.1155/2014/727451

**Published:** 2014-06-26

**Authors:** Pervez Sultan, Mark R. Edwards, Ana Gutierrez del Arroyo, David Cain, J. Robert Sneyd, Richard Struthers, Gary Minto, Gareth L. Ackland

**Affiliations:** ^1^Clinical Physiology, Division of Medicine, University College London, London WC1E 6BT, UK; ^2^Department of Anaesthesia, Derriford Hospital and Peninsula Medical School, Plymouth PL6 8DH, UK

## Abstract

Explanatory mechanisms for the association between poor exercise capacity and infections following surgery are underexplored. We hypothesized that aerobic fitness—assessed by cardiopulmonary exercise testing (CPET)—would be associated with circulating inflammatory markers, as quantified by the neutrophil-lymphocyte ratio (NLR) and monocyte subsets. The association between cardiopulmonary reserve and inflammation was tested by multivariable regression analysis with covariates including anaerobic threshold (AT) and malignancy. In a first cohort of 240 colorectal patients, AT was identified as the sole factor associated with higher NLR (*P* = 0.03) and absolute and relative lymphopenia (*P* = 0.01). Preoperative leukocyte subsets and monocyte CD14^+^ expression (downregulated by endotoxin and indicative of chronic inflammation) were also assessed in two further cohorts of age-matched elective gastrointestinal and orthopaedic surgical patients. Monocyte CD14^+^ expression was lower in gastrointestinal patients (*n* = 43) compared to age-matched orthopaedic patients (*n* = 31). The circulating CD14^+^CD16^−^ monocyte subset was reduced in patients with low cardiopulmonary reserve. Poor exercise capacity in patients without a diagnosis of heart failure is independently associated with markers of inflammation. These observations suggest that preoperative inflammation associated with impaired cardiorespiratory performance may contribute to the pathophysiology of postoperative outcome.

## 1. Introduction

Immune dysregulation is a key feature of low cardiac output states. Absolute numbers, as well as function, of monocytes and T-cells are markedly altered in cardiac failure [1]. Severe heart failure is associated with higher levels of circulating endotoxin [2] and lymphopenia [3]. Systemic inflammation driven by exposure to endotoxin in patients with heart failure results in downregulation of monocyte CD14^+^ expression and increased soluble CD14 through shedding of this receptor from the cellular membrane. Alterations in three distinct CD14^+^ monocyte subsets occur in various pathophysiological states, as defined by CD16 (Fc*γ*RIII) expression. The function of these subsets appears to be highly context-specific, with CD16^+^ subsets frequently expanded in chronic inflammatory conditions. Notably, in patients with chronic systolic heart failure, the nonclassical CD14^dim⁡^CD16^+^ subset is more prevalent and inversely associated with worsening cardiac performance [4]. By contrast, reduced levels of the classical CD14^++^CD16^−^ monocyte subset are reported in heart failure, compatible with remodelling roles for different subsets [5].

Poor exercise capacity in surgical patients, as measured objectively by preoperative cardiopulmonary exercise [6–12], is associated with poorer postoperative outcomes—including infection—following surgery [13]. Plausible mechanisms that explain the link between poor exercise capacity and poorer postoperative outcomes remain underexplored. Whether preoperative exercise capacity in surgical patients is similarly independently linked to markers of inflammation, as reported in heart failure, remains unclear. We therefore hypothesized that impaired cardiovascular performance (as defined by cardiopulmonary exercise testing) would be associated with biomarkers for inflammation (neutrophil-lymphocyte ratio and absolute and relative lymphopenia) and/or evidence consistent with surface markers indicative of changes in monocyte function by circulating endotoxin.

## 2. Materials and Methods

### 2.1. Patient Populations

Patients undergoing major elective colorectal surgery, including those who were screened for the COMPETE-C randomized controlled trial [14], underwent CPET at Derriford Hospital, Plymouth, UK, as approved by the Cornwall and Plymouth Research Ethics Committee (Medical Research Ethics Committee (MREC): 08/H0203/159). Full details of this trial (including CONSORT information) have been reported previously [14]. Further preoperative immune analysis was undertaken in two Research Ethics Committee approved studies at University College London Hospitals enrolling elective orthopaedic patients, healthy volunteers (MREC: 09/H0805/59), and patients undergoing major gastrointestinal surgery (MREC: 09/H0805/58). Adherence to STROBE guidelines is documented in Table S1 in Supplementary Material available online at http://dx.doi.org/10.1155/2014/727451.

### 2.2. Cardiopulmonary Exercise Testing

Patients completed symptom-limited maximal cardiopulmonary exercise testing (CPET) as part of their routine preoperative assessment. Following a period of 2 min rest and 2 min unloaded pedalling, patients began ramped, continuous incremental, and symptom limited exercise on a stationary ergometer (Zan, nSpire, CO, USA), with a ramp gradient 15–20 W/minute.

CPET was stopped as determined by the patients' tolerance. Ventilation and gas exchange variables were measured using a metabolic cart (Zan, nSpire, CO, USA). 12-lead electrocardiogram, noninvasive blood pressure, and pulse oximetry were monitored throughout the test period. Anaerobic threshold (AT) was used as the marker of aerobic fitness. AT was determined by two independent assessors according to the published guidelines using V-slope and confirmed by ventilatory equivalents [15].

*Experiment 1* (differential leukocyte counts). Preoperative blood samples were used to assess leukocyte subsets in both centres (Sysmex XE2100 analyzer, Sysmex, Milton Keynes, UK).

*Experiment 2* (flow cytometry assessment of monocytes). Heparinised blood samples were collected in heparin from preoperative patients at the same time of day, who had fasted for at least 6 h. All patients were undergoing elective surgery and were free from infection. Flow cytometry (Cyan ADP cytometer, Beckman Coulter, High Wycombe, UK) was performed using 100 *μ*L of freshly obtained whole blood samples placed on ice immediately after collection and processed in accordance with published guidelines [16]. Samples were processed within 2 h of collection. Control beads were used prior to each run. CD14^+^ expression was quantified in monocytes identified by forward and side scatter characteristics, combined with HLA-DR surface expression. Antibodies for CD14, CD16, and HLA-DR were purchased from Miltenyi Biotec, UK. Respective murine isotype controls (IgG2a and IgM, resp.; Miltenyi Biotec, UK) were used for each surface marker. Fc receptor blocker was used routinely. After incubation for 20 minutes, samples were fixed with 2% paraformaldehyde and red blood cells were lysed using isotonic ammonium chloride buffer. At least 10000 gated events were captured. Flow cytometry data were analyzed using Kaluza software (version 1.2; Beckman Coulter, High Wycombe, UK). Using an established gating strategy [17], three distinct subsets that are altered in various pathophysiological states of CD14^+^ monocyte subsets were identified by CD16 (Fc*γ*RIII) expression.

### 2.3. Statistics

To test the association between cardiopulmonary limitation and preoperative inflammation, multiple regression analysis (1-way hierarchical switching model) was undertaken with NLR as the dependent variable. AT was entered as a continuous independent variable with the following categorical independent variables: risk factors for cardiovascular disease [18], presence of malignancy, age, and gender. The relationship between a preoperative neutrophil-lymphocyte ratio >2.8—which is predictive of overall survival following colorectal surgery—and AT was also assessed (Fisher's exact test) [19]. For continuous data, variables were analysed with ANCOVA. Nonparametric data were analysed with the Mann-Whitney *U* test. All reported *P* values are two-sided. Statistical analyses were performed using NCSS 8 (Kaysville, UT, USA).

### 2.4. Sample Size Calculations

We powered the primary outcome (NLR) on the basis that ~30% colorectal patients with low AT (<11 mL*·*kg^−1^ *·*min^−1^) [13] would exhibit a prognostically significant difference in NLR ≥0.5 [20–23], with an anticipated standard deviation of 1.0. Thus, ≥185 patients with AT > 11 mL*·*kg^−1^ *·*min^−1^, compared with ≥56 patients with low AT (alpha = 0.05; power = 90%). Based on preceding studies in the cardiac failure literature measuring soluble CD14 [24], we sought to find an absolute difference in monocyte CD14 median fluorescence intensity of 25 arbitrary units (anticipated standard deviation of 25 arbitrary units), thus requiring a sample size of ≥32 cases per group (*α* = 0.01; power = 90%).

## 3. Results

**Figure 1:**
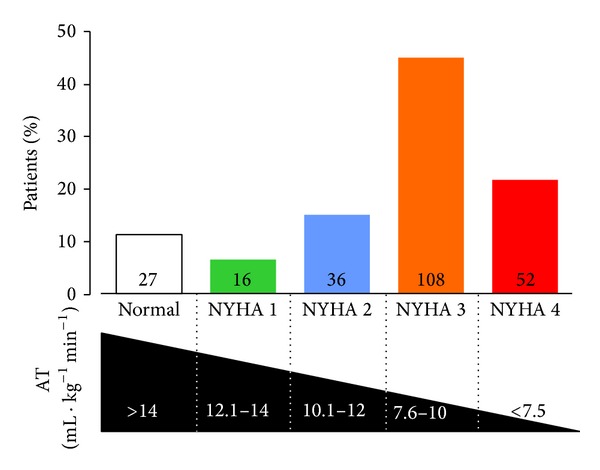
Histogram showing numbers of patients (*n* = 240) stratified by AT-defined NYHA class. Cutoff values for AT estimated from recent published series [25, 26].

**Figure 2:**
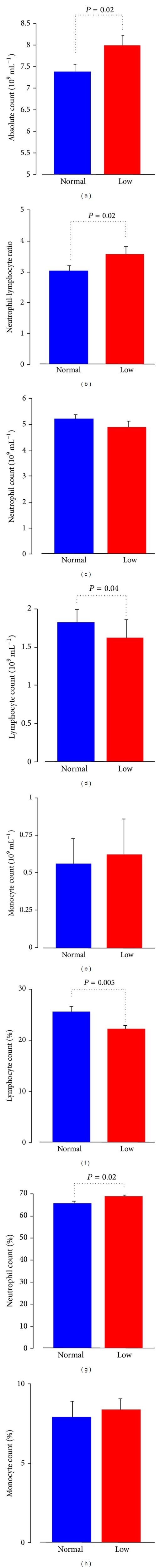
Leukocyte subsets according to AT value associated with poorer postoperative outcomes. (a) White cell count. (b) Neutrophil-lymphocyte ratio. (c) Absolute neutrophil count. (d) Absolute lymphocyte count. (e) Absolute monocyte count. (f) Proportion of lymphocytes. (g) Proportion of neutrophils. (h) Proportion of monocytes. All data are represented as mean ± SD; *n* = 240 patients.

*Experiment  1* (CPET physiological characteristics and leukocyte subsets). Demographics and associated cardiopulmonary test parameters of patients undergoing preoperative CPET are shown in Table 1. The majority of anaerobic threshold values were consistent with those reported for NYHA Classes 3-4 (Figure 1). Impaired cardiovascular performance was associated independently with higher NLR (*P* = 0.04) and absolute (*P* = 0.007) and relative lymphopenia (*P* = 0.004), adjusted for the presence of malignancy. Unadjusted for malignancy, low AT was associated with higher NLR (low AT: +0.54 (95% CI: 0.1–0.98); *P* = 0.01) and absolute (low AT: −0.20 lymphocytes 10^9^ mL^−1^ (95% CI: 0.01–0.40); *P* = 0.04) and relative lymphopenia (low AT: −3.4% (95% CI: 1.05–5.79); *P* = 0.005; Figure 2).

Controlling for the presence of malignancy (present in 77% of patients), low AT remained associated with higher NLR (low AT: +0.54 (95% CI: 0.1–0.98); *P* = 0.01) and absolute (low AT: −0.20 lymphocytes 10^9^ *·*mL^−1^ (95% CI: 0.01–0.40); *P* = 0.04) and relative (low AT: −3.4% (95% CI: 1.05–5.79); *P* = 0.005) lymphopenia, with no significant interaction observed between malignancy and AT < 11 mL*·*kg^−1^ *·*min^−1^ (*P* = 0.29). Multiple regression analysis identified AT as the sole factor associated with higher NLR (*P* = 0.033). An AT < 11 mL*·*kg^−1^ *·*min^−1^ was strongly associated with a preoperative NLR reported to be predictive of survival postoperatively (relative risk: 1.9 (95% confidence intervals: 1.4–2.5); *P* = 0.01) [19].

**Figure 3:**
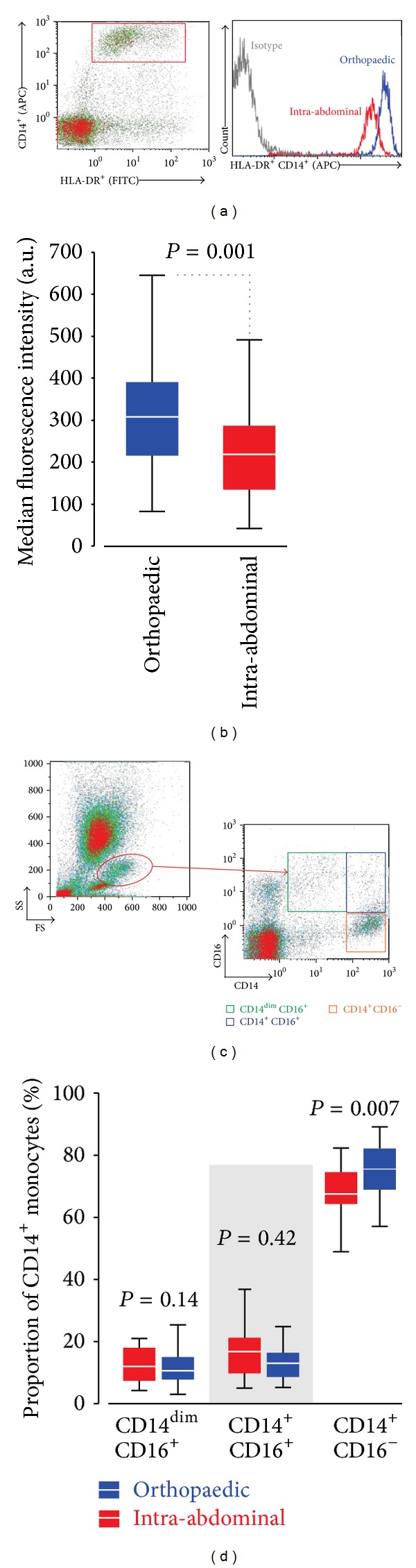
Monocyte CD14^+^ expression. (a) Representative HLA-DR^+^ CD14^+^ expression (median fluorescence intensity) in orthopedic (blue) and gastrointestinal (red) surgical patients. Isotype control shown in grey. (b) Summary data for orthopedic (*n* = 43 patients) and gastrointestinal malignancy (*n* = 31 patients). (c) Gating strategy to define HLA-DR^+^ CD14^+^monocyte subsets, including CD16^+^cells. (d) Proportions of monocyte CD14^+^ subsets according to CD16^+^ expression. All data are represented as median (IQR).

*Experiment  2* (preoperative monocyte CD14^+^ expression). Next, we assessed whether age-matched patients free of malignancy showed any evidence for systemic inflammation as indicated by biomarker levels found in patients undergoing colorectal surgery. We reasoned that monocyte CD14 surface expression would be reduced in patients with low AT, compared to age-matched controls with no clinical evidence for cardiac failure. Preoperative neutrophil-lymphocyte ratio was higher (*P* = 0.01), and monocyte CD14^+^ expression was lower (−112 median fluorescence units (95% CI: 49–176); *P* = 0.002) in 38 patients undergoing major surgery for gastrointestinal malignancy (median AT: 10 mL*·*kg^−1^ *·*min^−1^ (IQR 9–11)), compared to age-matched orthopaedic patients (*n* = 31) without overt clinical heart failure (Figures 3(a) and 3(b)). CD14^+^ monocyte subset analysis (Figure 3(c)) showed that the CD14^+^CD16^−^ subset was reduced in patients with low cardiopulmonary reserve (Figure 3(d)).

## 4. Discussion

Our data show that impaired cardiovascular performance as measured by CPET is associated with changes in leukocyte subsets that have emerged as robust prognostic markers of colorectal surgical outcome. Consistent with these data, we also found that downregulation of monocyte CD14^+^ expression and lymphopenia were associated with impaired cardiorespiratory reserve.

Maintenance, or enhancement, of exercise capacity has been associated with a range of beneficial immune responses [27]. Habitual exercise is associated with improved immune responses to vaccination [28] and outcomes following viral infections [29] and malignancy [30]. Immune dysregulation is a pivotal feature of poor aerobic capacity [1], with the most severe grades of heart failure associated with higher levels of circulating endotoxin [2] and lymphopenia [3]. Our data suggest that in higher-risk surgical patients similar immune dysregulation may occur, even though such patients have never received a formal diagnosis of cardiac failure. Since the presence of overt cardiac failure is an important prognostic factor in determining postoperative outcome [31], our data also suggest that dysregulation of inflammation is associated with poor cardiopulmonary reserve in preoperative patients. Preexisting inflammation could contribute to increased morbidity and mortality after surgery—in addition to any putative mechanistic role for impaired perioperative cardiovascular performance. Interestingly, several studies have reported an association between preoperative NLR and postoperative complications [19–23] but have not made the link with another strong predictor of postoperative morbidity—aerobic performance. Taken together, these data suggest that low AT may therefore be a marker, rather than a direct mediator, of poorer perioperative outcomes.

Experimental and clinical data suggest that established immune dysfunction is instrumental in driving impaired cardiovascular function, rather than merely being a marker of disease progression [1]. Thus, targeting immune function is an increasingly important line of investigation in efforts to reduce the substantial mortality from the increasing prevalence of cardiac failure. Strikingly, similar data from the perioperative literature supports this concept: patients with evidence for preoperative depletion of antibodies to endotoxin exhibit higher levels of proinflammatory cytokines and sustain more perioperative morbidity [32, 33]. Downregulation of CD14^+^ is consistent with the hypothesis that chronic systemic exposure to low plasma levels of endotoxin occurs as a result of (occult) cardiac failure [34, 35]. Alternatively, these data may reflect gastrointestinal pathology and endotoxin “leak” [36]. Nevertheless, our results are consistent with previous studies reporting that the CD14^++^CD16^−^ monocyte subset was reduced in patients with chronic systolic heart failure [34].

Monocyte-macrophage function is altered by exercise-induced activation of both sympathetic and hypothalamic-pituitary axes [37]. Absolute monocyte count transiently increases in response to acute exercise, accompanied by preferential demarginalization of the proinflammatory CD14^+^CD16^+^ subset [38] and downregulation of Toll-like receptors [39]. After cessation of exercise, the CD14^+^CD16^+^ subset declines—indicative of remarginalization or recruitment to tissue [40]. Sustained exercise is associated with reduced circulating monocyte inflammatory responses to lipopolysaccharide, lower TLR4 expression, and a lower percentage of the proinflammatory CD14^+^CD16^+^ [41]. Human data regarding the impact of chronic exercise on, as well as tissue resident function of, monocyte function is limited. However, murine models suggest that exercise-trained mice demonstrate enhanced splenic macrophage proliferative capacity and peritoneal macrophages phagocytic activity [42]. Patients with more advanced heart failure, as reflected by low AT, demonstrate an increased number of neutrophils. This relative neutrophilia is accompanied by a decreased number of lymphocytes, characterised by higher proportions of terminally differentiated CD4^+^ and CD8^+^ T-subsets [43]. These data are consistent with molecular mechanisms driving a T-cell immunosenescent state [44].

We acknowledge several limitations of this exploratory study. Further detailed analyses of leukocyte subset functionality stratified by AT could be instructive; in particular, CD14^+^ subsets associated with an augmented proinflammatory phenotype or different migratory properties require further elucidation. Measurement of endotoxin levels would add further insight. The association between NLR and coronary artery disease [45] may reveal mechanistic links to perioperative outcomes, including myocardial injury. Ultimately, detailed outcome data—including propensity to infectious complications—will test the hypothesis further that low AT may be associated with defective innate and/or adaptive immune functionality.

In summary, impaired cardiovascular performance is associated with changes in leukocyte subsets that have emerged as robust prognostic markers of colorectal surgical outcome. Consistent with these data, we also find that downregulation of monocyte CD14^+^ expression and lymphopenia are associated with impaired cardiorespiratory reserve. These observations suggest that preoperative inflammation, associated with impaired cardiorespiratory performance, may contribute a pathophysiological role in determining postoperative outcome.

## Supplementary Material

STROBE Statement—Checklist of items that should be included in reports of cohort studies.

## Figures and Tables

**Table 1:** CPET demographics in colorectal surgery cohort, stratified according to the prognostically relevant anaerobic threshold <11 mL kg^−1^ min^−1^ and >11 mL kg^−1^ min^−1^.

	*V*_AT_ < 11 mL kg^−1^ min^−1^	*V*_AT_ > 11 mL kg^−1^ min^−1^	*P* value
Number	79	161	
Age (y)	71 (69–73)	63 (61–66)	0.0001
Gender (male : female ratio)	1.05	1.47	0.30
Cancer (*n*; %)	66 (86%)	114 (72%)	0.02
Chemotherapy (*n*; %)	21 (13%)	11 (14%)	0.82
Body mass index (kg m^2^)	29.6 (28.5–30.6)	27.4 (26.7–28.0)	0.0004
${\dot{\text{V}}}_{\text{E}}$ /$\dot{\text{V}}$CO_2_	30.3 (28.9–31.7)	27.8 (27.2–28.5)	0.0004
Peak $\dot{\text{V}}$O_2_ (mL kg^−1^ min^−1^)	14.8 (13.9–15.6)	22.5 (21.5–23.4)	<0.0001

Data are presented as mean (95% confidence intervals), unless stated otherwise.

## Abbreviations

AT: Anaerobic threshold.
